# Visualization of tumor heterogeneity and prediction of isocitrate dehydrogenase mutation status for human gliomas using multiparametric physiologic and metabolic MRI

**DOI:** 10.1038/s41598-022-05077-2

**Published:** 2022-01-20

**Authors:** Akifumi Hagiwara, Hiroyuki Tatekawa, Jingwen Yao, Catalina Raymond, Richard Everson, Kunal Patel, Sergey Mareninov, William H. Yong, Noriko Salamon, Whitney B. Pope, Phioanh L. Nghiemphu, Linda M. Liau, Timothy F. Cloughesy, Benjamin M. Ellingson

**Affiliations:** 1 UCLA Brain Tumor Imaging Laboratory (BTIL), Center for Computer Vision and Imaging Biomarkers, University of California, Los Angeles, 924 Westwood Blvd., Suite 615, Los Angeles, CA 90024 USA; 2 Department of Radiological Sciences, David Geffen School of Medicine, University of California, Los Angeles, Los Angeles, CA USA; 3 Department of Radiology, Juntendo University School of Medicine, Tokyo, Japan; 4 Department of Diagnostic and Interventional Radiology, Osaka City University Graduate School of Medicine, Osaka, Japan; 5 Department of Bioengineering, Henry Samueli School of Engineering and Applied Science, University of California Los Angeles, Los Angeles, CA USA; 6 Department of Neurosurgery, David Geffen School of Medicine, University of California, Los Angeles, Los Angeles, CA USA; 7 Department of Pathology, David Geffen School of Medicine, University of California Los Angeles, Los Angeles, CA USA; 8 UCLA Neuro-Oncology Program, University of California, Los Angeles, Los Angeles, CA USA; 9 Department of Neurology, David Geffen School of Medicine, University of California Los Angeles, Los Angeles, CA USA; 10 Department of Psychiatry and Biobehavioral Sciences, David Geffen School of Medicine, University of California Los Angeles, Los Angeles, CA USA

**Keywords:** Biomarkers, Predictive markers

## Abstract

This study aimed to differentiate isocitrate dehydrogenase (IDH) mutation status with the voxel-wise clustering method of multiparametric magnetic resonance imaging (MRI) and to discover biological underpinnings of the clusters. A total of 69 patients with treatment-naïve diffuse glioma were scanned with pH-sensitive amine chemical exchange saturation transfer MRI, diffusion-weighted imaging, fluid-attenuated inversion recovery, and contrast-enhanced T1-weighted imaging at 3 T. An unsupervised two-level clustering approach was used for feature extraction from acquired images. The logarithmic ratio of the labels in each class within tumor regions was applied to a support vector machine to differentiate IDH status. The highest performance to predict IDH mutation status was found for 10-class clustering, with a mean area under the curve, accuracy, sensitivity, and specificity of 0.94, 0.91, 0.90, and 0.91, respectively. Targeted biopsies revealed that the tissues with labels 7–10 showed high expression levels of hypoxia-inducible factor 1-alpha, glucose transporter 3, and hexokinase 2, which are typical of IDH wild-type glioma, whereas those with labels 1 showed low expression of these proteins. In conclusion, A machine learning model successfully predicted the IDH mutation status of gliomas, and the resulting clusters properly reflected the metabolic status of the tumors.

## Introduction

WHO classification of Tumors of the Central Nervous System was revised in 2016 to incorporate the molecular status, such as isocitrate dehydrogenase (IDH) gene mutation and chromosomal 1p/19q codeletion, for diagnosing diffuse gliomas^1^. IDH mutation is one of the most critical molecular markers and has considerable prognostic and predictive value, with the IDH mutant subtype showing a better prognosis and sensitivity to treatment^2^. Further, IDH mutation status has been proven to be superior to WHO grading in predicting the prognosis of glioma^3^. Thus, prompt and noninvasive prediction of IDH status is desired and would be more valuable as IDH inhibitors become a neoadjuvant therapy for IDH mutant gliomas^4^.

Physiologic and metabolic MRI have contributed to better understanding of tumor biology related to IDH mutation and prediction of IDH mutation status. Apparent diffusion coefficient (ADC), obtained from diffusion-weighted imaging (DWI), is an estimate of the random motion of water molecules, and a strong negative correlation between the ADC and tumor cellularity has been shown^5^. Amine chemical exchange saturation transfer echo-planar imaging (CEST-EPI) is an MRI technique sensitive to tissue pH^6^. The proton exchange between amines and bulk water, which is detected by amine CEST, is a base-catalyzed process; thus, the exchange rate is dependent on pH^7^. ADC and amine CEST-EPI have been proven helpful in differentiating the IDH status of diffuse gliomas^8–10^. However, used alone, these metrics showed only moderate prediction ability of IDH mutation status, with the area under the curve (AUC) less than 0.9.

Machine learning with and without radiomics analysis has been used to extract numerous features that are not discernible to human eyes to fully exploit multiparametric MRI data^11,12^. A number of studies have combined multiparametric MRI and machine learning to predict IDH status, while revealing specific imaging features related to IDH status^13,14^. However, the association of extracted textural features with histology has been elusive^15^. Habitat imaging, which divides multiparametric imaging into distinctly different segments, can provide unique insights into associations between multiparametric MRI and biological subpopulations, or habitats, of a tumor^16^. Therefore, the purpose of this study is twofold: (1) to develop a voxel-wise clustering method using multiparametric MRI to predict IDH status and (2) to explore the association between the created cluster labels and immunohistochemical markers of glucose metabolism.

## Results

We reviewed the data of 159 patients with pathologically confirmed diffuse glioma that underwent CEST-EPI and DWI or DTI. The data of 90 patients were excluded from the study because the gliomas were treated prior to the scans. None of the patients had severe artifacts to be excluded. Thus, a total of 69 patients (47 men, median age, 53 years; range 19–80 years) were eligible for this study. Out of 69 diffuse gliomas, 32 were IDH mutant (14 were 1p/19q codeleted and 18 were 1p/19q non-codeleted) and 37 were IDH wild-type. Of the included patients, 14 underwent 45 biopsies. The detailed patient characteristics are further outlined in Supplementary Table 1.

The number of K-classes that showed the best performance for predicting IDH mutation status was explored by comparing AUC, accuracy, and F1 score among different K-classes using a 100-bootstrap sampling. The 10-class clustering showed the highest AUC and F1 score, significantly higher than the K-classes 4, 6, 8, and 12, and non-significantly higher than the K-classes 16 and 20 (Supplementary Fig. 1). The 10-class clustering showed the highest accuracy, significantly higher than the K-classes 4, 6, 8, 12, and 20, and non-significantly higher than the K-class 16 (Supplementary Fig. 1). The prediction performance for all K-classes is summarized in Supplementary Table 2. The mean and 95% confidence interval of the AUC, accuracy, sensitivity, specificity, precision, recall, and F1 score of K-clustering = 10 were 0.94 [0.94–0.95], 0.91 [0.90–0.92], 0.90 [0.89–0.91] 0.91 [0.90–0.92], 0.90 [0.89–0.91], 0.90 [0.89–0.91], and 0.90 [0.89–0.91], respectively. When age was included in the SVM analysis, the mean and 95% confidence interval of AUC, accuracy, sensitivity, specificity, precision, recall, and F1 score were 0.94 [0.93–0.94], 0.90 [0.89–0.90], 0.89 [0.87–0.90], 0.90 [0.89–0.91], 0.88 [0.87–0.89], 0.89 [0.87–0.90], and 0.88 [0.87–0.89], respectively. In the following sections, only the results related to K = 10 will be shown.

**Figure 1:**
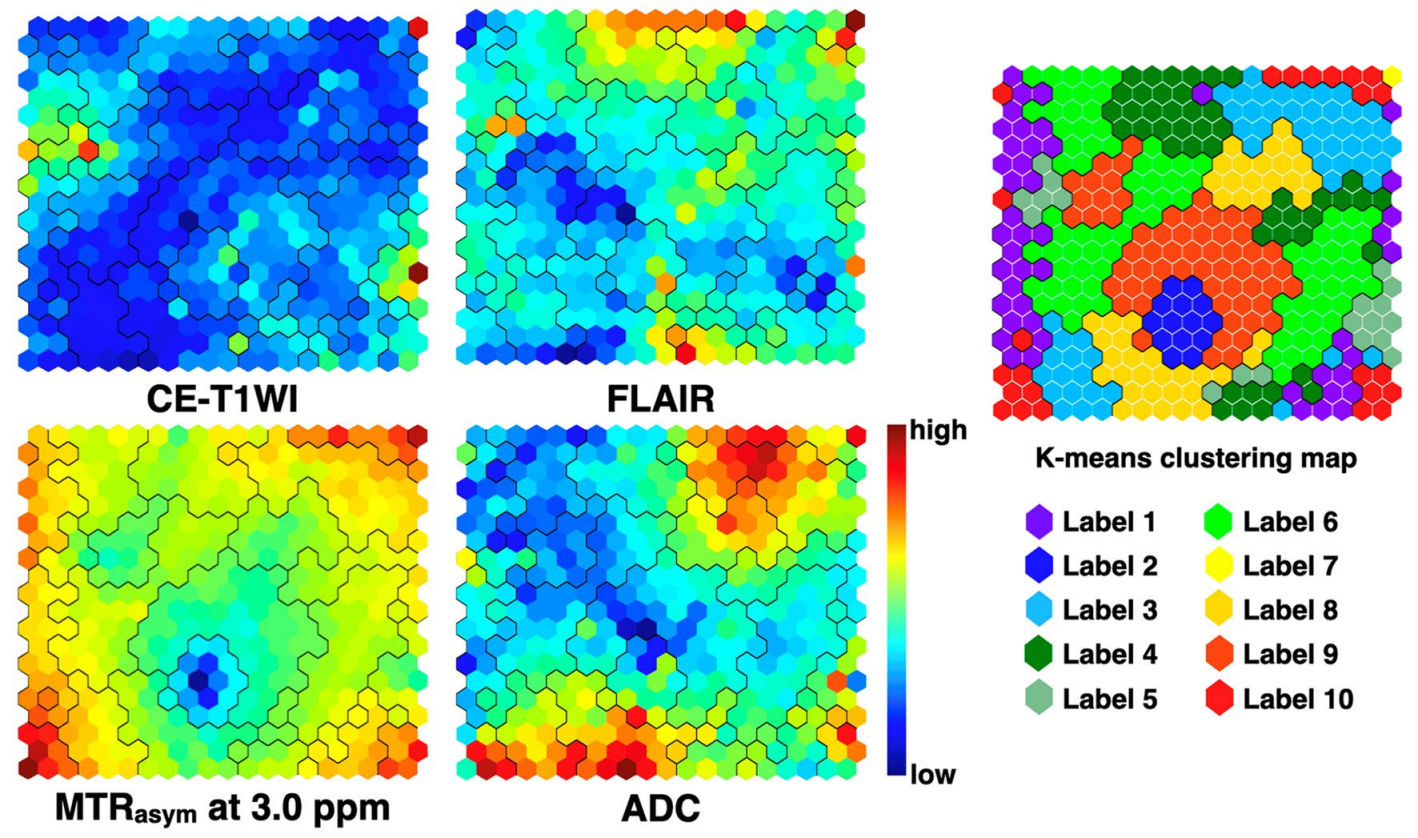
Component planes with a SOM for CE-T1WI, FLAIR, MTR_asym_ at 3.0 ppm, and ADC colorized from blue to red according to each value, with red indicating a higher weight. The inter-class borderlines obtained by K-means clustering with K = 10 are shown on the SOM component planes as black lines between the nodes. Detailed profiles can be seen on the K-means clustering map from labels 1 to 10 shown at the far right.

The component planes of the four variables from contrast-enhanced T1-weighted images (CE-T1WI), fluid-attenuated inversion recovery (FLAIR), asymmetric magnetization transfer ratio (MTR_asym_) at 3.0 ppm, and ADC by the self-organizing map (SOM) analysis showed the information of each sequence in each map unit as well as the associations between the protoclusters and each image (Fig. 1). The component planes of the four variables differed largely from each other, indicating that these variables contain unique information. These protoclusters were classified into 10 labels by K-means (K = 10).

**Figure 2:**
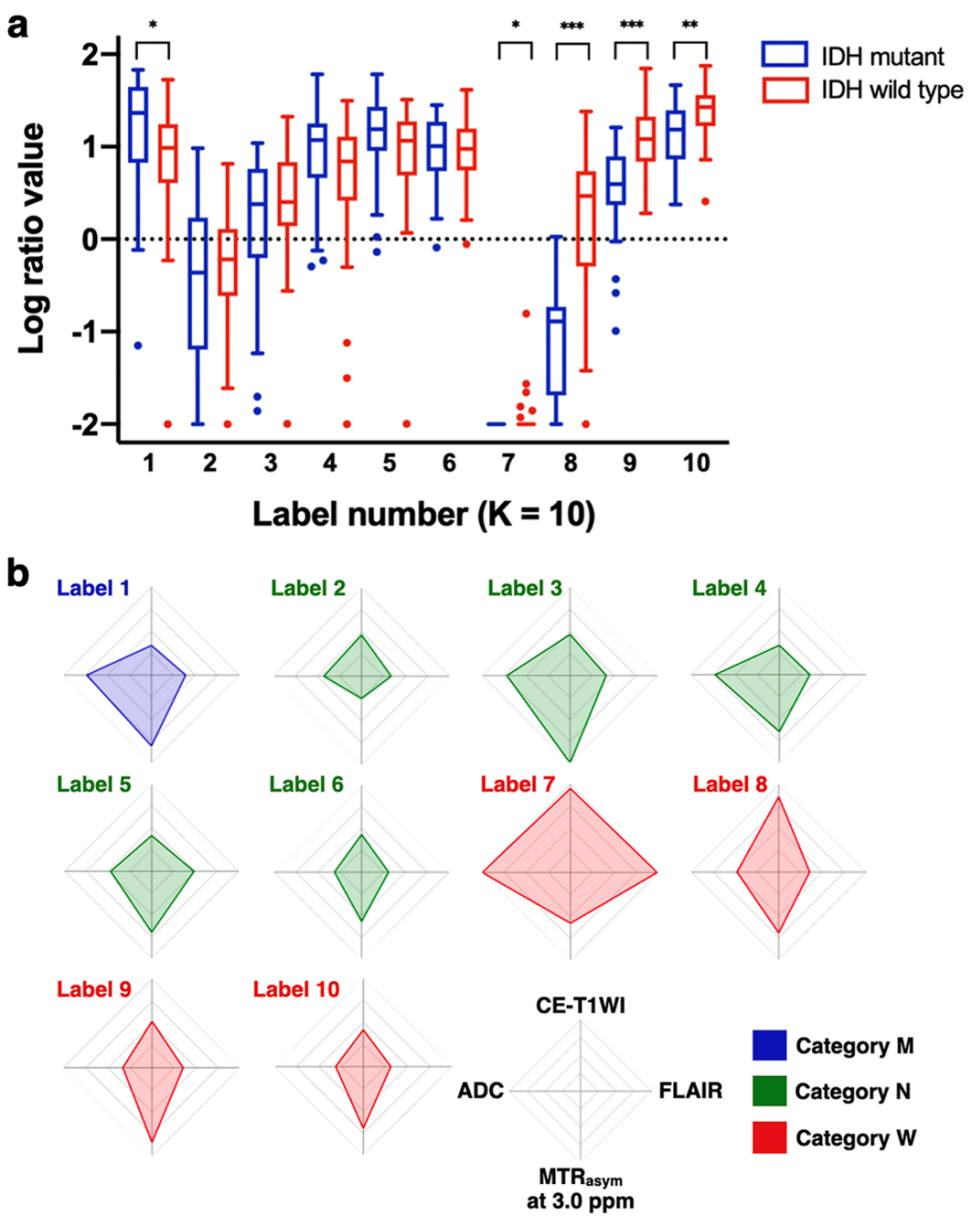
(**a**) Box-whisker plots and (**b**) radar charts of labels by 10-class clustering. (**a**) The box shows the interquartile range between the 25th and 75th percentiles for log-ratio values; the lines within the boxes represent medians, and the whiskers represent measurements 1.5 times the interquartile range. The circles represent outliers beyond 1.5 times the interquartile range. * P < 0.05, ** P < 0.01, *** P < 0.001. (**b**) Radar charts of four variables (CE-T1WI, FLAIR, MTR_asym_ at 3.0 ppm, and ADC) in each label categorized into three groups.

**Figure 3:**
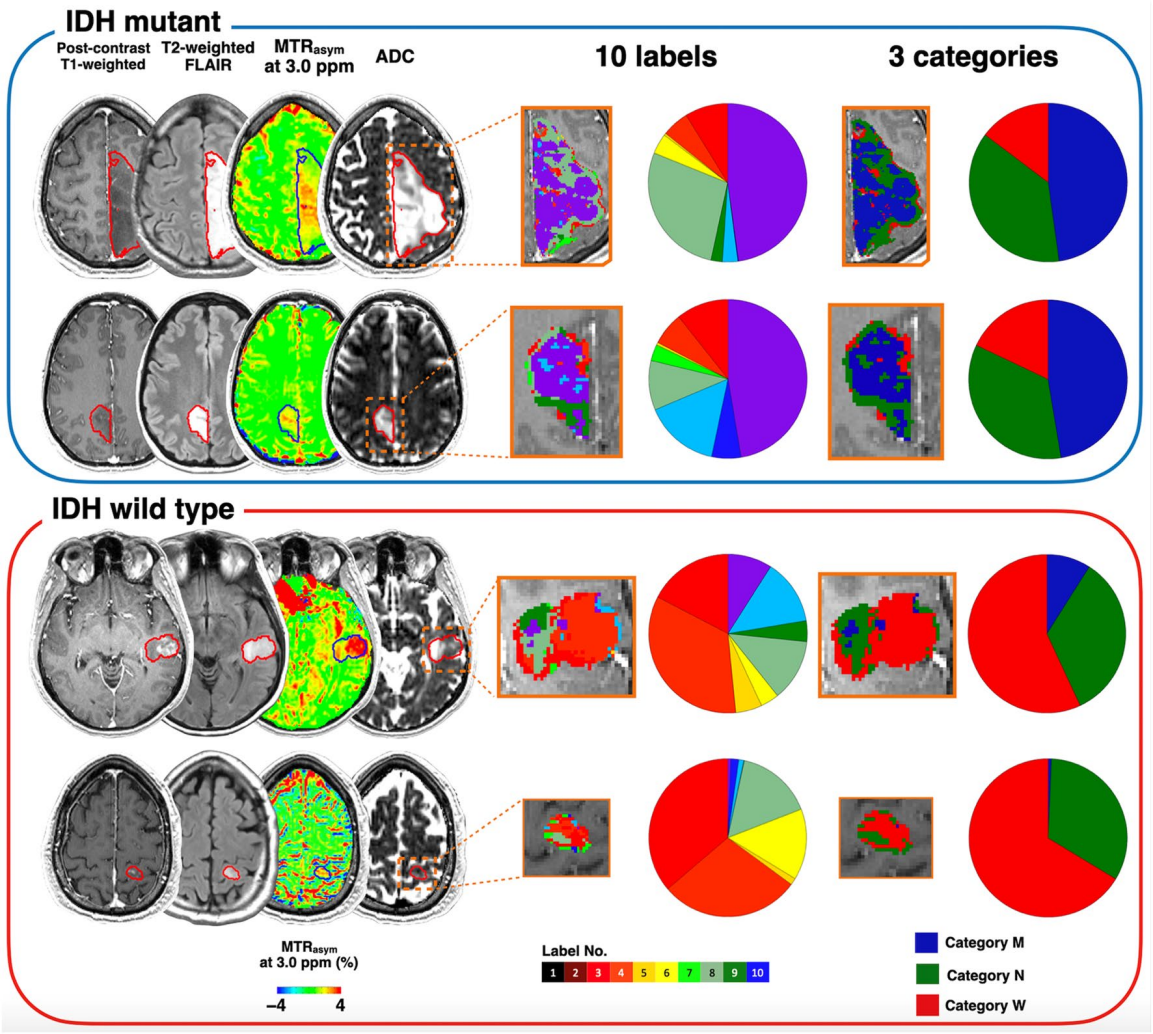
Representative cases of IDH mutant and wild-type gliomas with 10-class clustering. The CE-T1WI and FLAIR images, MTR_asym_ at 3.0 ppm, and ADC maps are shown for each patient. Each color within the tumor ROIs corresponds to each label in the 10-color bar and each category in the 3-color bar. The ratios of each label and category are shown in pie charts. In these examples, labels in category *M* (label 1) occupied about half of the tumor ROIs in IDH mutant gliomas, while labels in category *W* (label 7–10) occupied less than a quarter of the tumor ROIs. In IDH wild-type gliomas, labels in category *W* occupied the majority of the tumor ROIs.

The log-ratio values of each label in the K = 10 class were compared between IDH mutant and wild-type gliomas (Fig. 2a). The log-ratio value of label 1 was significantly higher in IDH mutant than in wild-type gliomas (P < 0.05), and these labels were categorized as *M*. In contrast, the log-ratio values of labels 7–10 were significantly higher in IDH wild-type than in mutant gliomas (P < 0.05, P < 0.001, P < 0.001, and P < 0.01, respectively), and these labels were categorized as *W*. Other labels (2–6) were categorized as N. The radar charts of the individual normalized values of the four images for each label in the K = 10 class are shown in Fig. 2b. Labels 7–9 in category *W* showed higher CE-T1WI values than other labels. Label 9 in category *W* showed a higher MTR_asym_ at 3.0 ppm than other labels, except for label 3 in category *N*. The labels 9 and 10 in category *W* showed lower ADCs than other labels. In contrast, label 1 in category *M* showed the highest ADC. FLAIR did not show a clear trend. Figure 3 shows representative cases of IDH mutant and wild-type gliomas.

**Figure 4:**
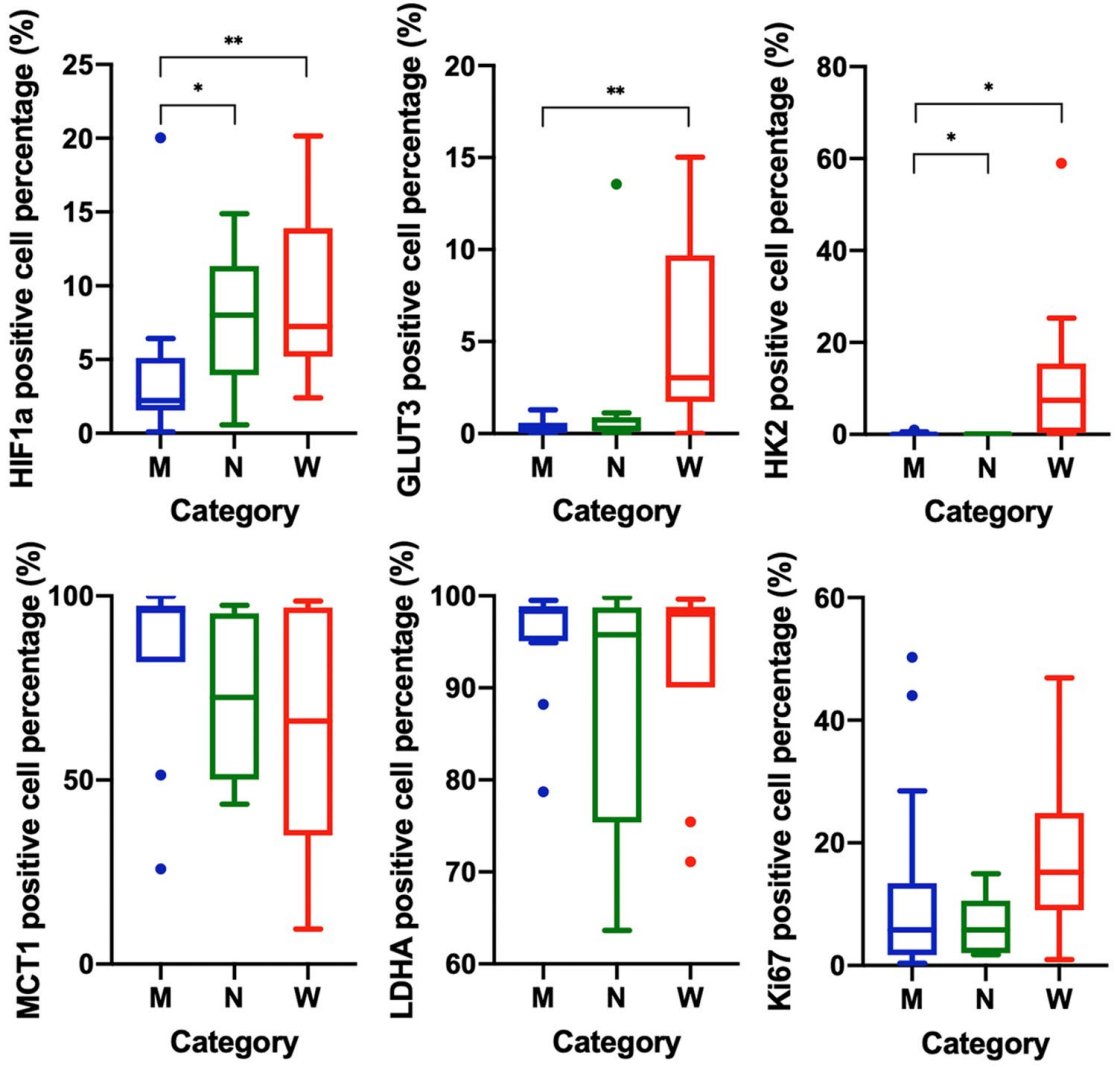
Box-whisker plots of histological measurements, namely, HIF1a-, GLUT3-, HK2-, MCT1-, LDHA-, and Ki67-positive cell percentages. The box shows the interquartile range between the 25th and 75th percentiles for positive cell percentage; the lines within boxes represent medians, and the whiskers represent measurements 1.5 times the interquartile range. The circles represent outliers beyond 1.5 times the interquartile range.

**Figure 5:**
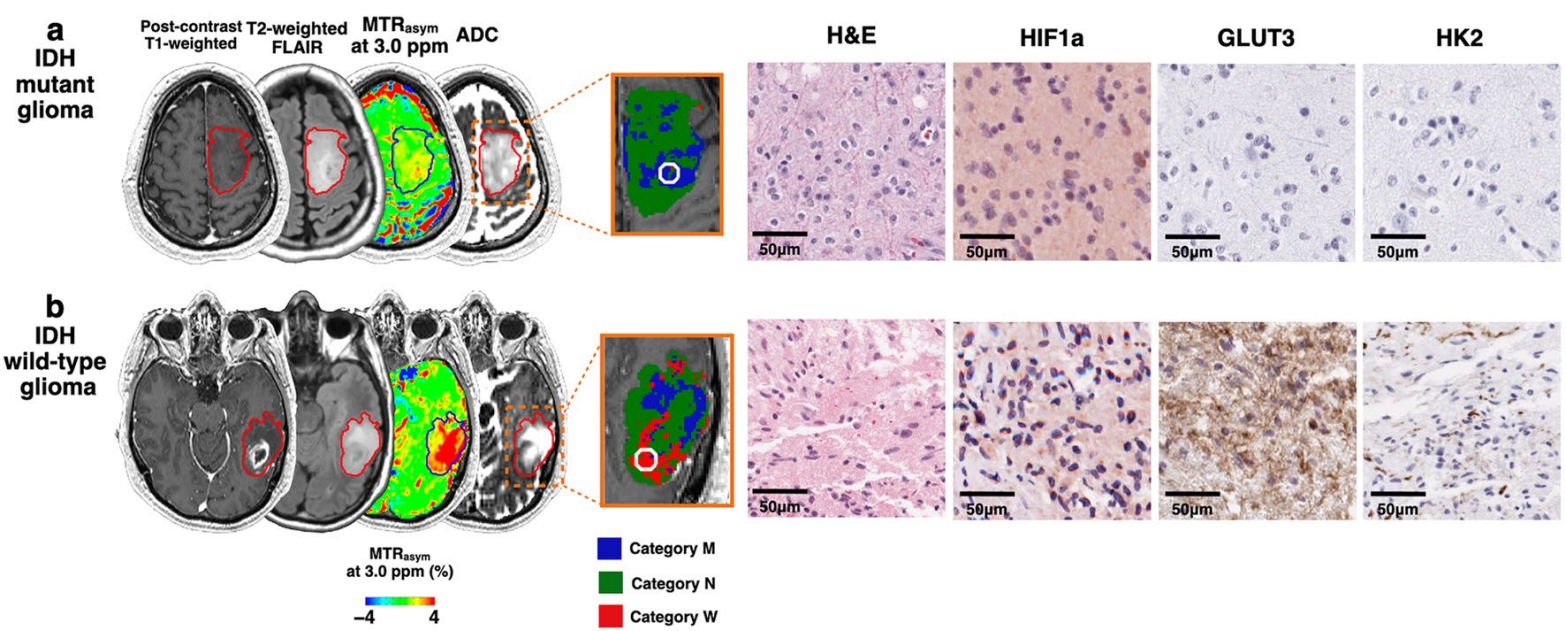
MR images and corresponding hematoxylin and eosin (H&E) and immunohistochemistry staining for MRI-guided biopsy targets (circles). (**a**) IDH mutant glioma for which an area with labels categorized as *M*, indicating the IDH mutant feature, was biopsied. Expressions of HIF1a, GLUT3, and HK2 are low in the slides from a 5-mm radius sample taken from the MRI-guided biopsy target. (**b**) IDH wild-type glioma for which an area with labels categorized as *W*, indicating IDH wild-type feature, was biopsied. Expressions of HIF1a, GLUT3, and HK2 are high.

The results of histological measurements on biopsy specimens are summarized in Fig. 4. hypoxia-inducible factor 1-alpha (HIF1a)-positive cell percentage was significantly higher in category *W* and *N* than in category *M* (9.51% ± 5.68% and 7.57% ± 4.53% vs. 3.83% ± 4.71%; mean ± standard deviation; P < 0.01 and < 0.05, respectively). glucose transporter 3 (GLUT3)-positive cell percentage was significantly higher in category *W* than in category *M* (5.54% ± 5.21% vs. 0.37% ± 0.44%; P < 0.01). hexokinase 2 (HK2)-positive cell percentage was significantly higher in category *W* than in categories *M* and *N* (12.94% ± 17.85% vs. 0.18% ± 0.28% and 0.10% ± 0.10%; both P values < 0.05). monocarboxylate transporter 1 (MCT1)-, lactic dehydrogenase A (LDHA)-, and Ki67-positive cell percentages did not differ between categories *M*, *N*, and *W*. No comparison of histological measurements in each category between IDH mutant and wild-type gliomas was significant. When compared between IDH mutant and wild-type gliomas based on their pathological diagnoses, without considering MRI labels, HIF1a-positive cell percentage, GLUT3-positive cell percentage, and HK2-positive cell percentage were significantly higher in IDH wild-type than in IDH mutant gliomas (9.51% ± 7.11% vs. 3.84% ± 3.38%, P < 0.01 for HIF1a; 3.28% ± 4.61% vs. 1.17% ± 3.32%, P < 0.05 for GLUT3; and 7.23% ± 14.54% vs. 0.14% ± 0.25%, P < 0.05 for HK2). MCT1-, LDHA-, and Ki67-positive cell percentages did not differ between IDH mutant and wild-type gliomas. Figure 5 shows representative cases of IDH mutant and wild-type gliomas with MRI, biopsy targets, and histological measurements.

## Discussion

In this study, we developed a voxel-wise clustering method to predict and visualize IDH mutation status using multiparametric MRI, including CE-T1WI, FLAIR, MTR_asym_ at 3.0 ppm, and ADC, using an unsupervised two-level clustering approach. The performance was evaluated using SVM with LOOCV. This clustering method enabled visualization of the association of different imaging modalities in each cluster. Ten-class clustering showed the highest performance to predict IDH status, with AUC, accuracy, sensitivity, and specificity of 0.94, 0.91, 0.90, and 0.91, respectively. The accuracy of our study was higher than 0.78–0.90 as reported in previous studies that used only internal validation, such as n-fold cross validation and LOOCV, and included grade II–IV gliomas^17–21^. However, direct comparison of performance is not appropriate because machine learning analysis without a separate test set is insufficient to derive true performance. In addition to conventional MRI sequences, about half of the machine learning studies reported in two recent meta-analyses for predicting IDH status used advanced MRI sequences, such as DWI, diffusion tensor imaging (DTI), dynamic susceptibility contrast MRI, and functional MRI, and two studies used ^18^F-fluoroethyl-l-tyrosine positron emission tomography^14,22^. To our knowledge, the current study is the first to use CEST as a part of a machine-learning algorithm to predict IDH mutation status. The high performance of our algorithm, along with the visualization of tumor characteristics, merits further investigation using external validation datasets. The results of our study may not necessarily hold after external validation. Notably, the inclusion of age as an input in this study did not improve the performance. Age might have served as a redundant feature.

The key strength of this study is the visualization of topological associations between imaging parameters to predict IDH mutation status. This may help prioritize modalities in multiparametric images. CE-T1WI showed higher values in labels 7–9, categorized as W, than in other labels. Thus, contrast enhancement contributed to the prediction of IDH wild-type status. In contrast, FLAIR did not show such a clear trend. MTR_asym_ at 3.0 ppm showed the highest value in label 3, which was categorized as N and thus nonspecific. However, MTR_asym_ at 3.0 ppm showed the second-highest value in label 9, categorized as *W*. This is consistent with a previous study that showed higher MTR_asym_ at 3.0 ppm in IDH wild-type than IDH mutant gliomas^8^. The higher MTR_asym_ at 3.0 ppm can be attributed to increased expression of glycolysis-related genes in IDH wild-type glioma, which leads to increased production of lactate and resulting acidity^23^. Label 9 and 10, categorized as *W*, showed lower ADC than other labels, whereas label 1, categorized as *M*, showed the highest ADC among all labels. This is consistent with previous studies that showed lower ADC in IDH wild-type than IDH mutant glioma^10,24^. Even though ADC differences in gliomas have been attributed to tumor cellularity, necrosis, cyst, and interstitial water^25,26^, the lower ADCs in label 9 and 10 and higher ADC in label 1 are probably mainly due to the differences in cellularity.

Histological measurements revealed higher expression of HIF1a, GLUT3, and HK2 in labels in category *W* than in category *M*, which corresponded with the comparison between IDH wild-type and mutant gliomas based on pathological diagnoses. These results are in line with previous biochemical studies showing higher expression of HIF1a, GLUT3, and HK2 in IDH wild-type than mutant gliomas^27,28^. These proteins play critical roles in initiating and maintaining the high glycolytic rates of rapidly proliferating glioma cells and are associated with the malignant features of IDH wild-type gliomas^29^. Moreover, in the current study, no significant difference in histological measurement was found in all categories between IDH mutant and wild-type gliomas. This indicates that our clustering method properly categorized tumor parts into those with features of IDH mutant, wild-type, or neither, and this categorization was congruent with the metabolic features associated with glycolysis.

This study had some limitations. First, we used LOOCV instead of an external validation dataset to evaluate the performance of our algorithm. Notably, a recent study used only 3 K-means labels for ADC and normalized cerebral blood volume maps to avoid overparameterization in predicting survival of patients with glioblastoma^30^. There is a possibility that our 10 labels algorithm has resulted in overparameterization. External validation is required to ensure the generalizability of our method^31^. Second, the acquisition parameters and scanners were not identical for all participants in this study. However, these variabilities were mitigated by normalization of signal intensity/quantitative value normalization. Moreover, the diversity in acquisition parameters and scanners may have contributed to the generalizability of our method, although this should be confirmed with an independent external dataset. Third, limited by the size of the overall population, the classification performance to differentiate either IDH mutant 1p/19q codeleted (14/69) and non-codeleted (18/69) tumors from other subtypes was not reliable (differentiation of IDH mutant 1p/19q non-codeleted, F1-score 0.49; differentiation of IDH mutant 1p/19q non-codeleted, F1- score 0.48; other detailed data not shown); hence, we combined these two groups as IDH mutant gliomas. However, gliomas with different 1p/19q codeletion statuses seem to have specific imaging features, such as a lower MTR_asym_ at 3.0 ppm and ADC in 1p/19q codeleted gliomas than in non-codeleted gliomas^32,33^. Therefore, future research is warranted to predict 1p/19q codeletion status using our algorithm with a larger cohort. In conclusion, an unsupervised two-level clustering approach enabled prediction of the IDH mutation status of diffuse gliomas by using multiparametric MRI data. Moreover, the resulting clustered features enabled the depiction of voxel-wise image associations and their relationship with immunohistochemical metabolic markers.

## Materials and methods

### Patient selection

This study was conducted with institutional review board approval (IRB# 14-001261; 10-000655). Written informed consent was acquired from all participants prior to study-related procedures. All de-identified patient information was stored on a secure research database. We reviewed the data of patients with pathologically confirmed diffuse glioma that underwent CEST-EPI and DWI or DTI between April 2015 and October 2019. The exclusion criteria were prior treatment and severe artifacts. IDH mutation status, including both IDH1 and IDH2 mutations, was confirmed by genomic sequencing analysis using IHC, polymerase chain reaction, or both as previously described^34^. 1p/19q codeletion status was assessed using fluorescence in situ hybridization.

### MR acquisition

All patients were scanned with CEST-EPI (single echo) or CEST spin-and-gradient-echo EPI (CEST-SAGE-EPI), DWI or DTI, and anatomical imaging on 3-T scanners (Prisma, Skyra, or Trio, Siemens Healthcare, Erlangen, Germany). Anatomical imaging was performed according to the standardized brain tumor imaging protocol^35^. CEST imaging, DWI, and DTI were performed before contrast administration. The CEST-SAGE-EPI sequence consisted of a saturation pulse train of three 100-ms Gaussian pulses with the peak amplitude B_1_ of 6 μT and a SAGE-EPI readout consisting of 2 gradient echoes with echo times (TEs) of 14.0 and 34.1 ms, one asymmetric spin-echo with a TE of 58.0 ms, and one spin-echo with a TE of 92.4 ms. The other acquisition parameters were as follows: repetition time (TR) > 10,000 ms; field of view = 217 × 240 mm; matrix size = 116 × 128; number of slices = 25; slice thickness = 4.0 mm with no interslice gap; partial Fourier encoding = 6/8, generalized autocalibrating partially parallel acquisition = 3; and bandwidth = 1628 Hz/pixel. A total of 29 z-spectral points were acquired at offset frequencies from − 3.5 ppm to − 2.5 ppm, − 0.3 ppm to + 0.3 ppm, and + 2.5 ppm to + 3.5 ppm, all with respect to the water proton resonance frequency. An additional reference (S0) scan was obtained with four averages using identical parameters without saturation pulses. The other details of the sequence are described elsewhere^36^. Some of the participants were scanned with a single-echo pH-weighted CEST-EPI sequence^6^ with a TE of 27 ms. The total acquisition time of CEST-SAGE-EPI was 7 min 30 s, benchmarked on a 3-T Prisma MR scanner (Software Version VE11C).

DWI using a single-shot echo-planar sequence or DTI along 64 motion-probing gradients was performed with the following parameters: b = 1000 s/mm^2^ and 0 s/mm^2^, TR/TE = 10,000/108 ms for DWI and 4100–6000/71–84 ms for DTI; field of view = 240 × 240 mm for DWI and 256 × 256 for DTI; matrix size = 128 × 128; slice thickness = 3 mm for DWI and 2 mm for DTI with no interslice gap; number of slices = 52 for DWI and 72 for DTI; and acquisition time of approximately 3 min for DWI and 5–7 min for DTI. ADC maps were calculated from the DWI and DTI data.

### Postprocessing of MRI data

CEST-EPI and CEST-SAGE-EPI data were used to calculate the MTR_asym_ at amine proton resonance frequency (3.0 ppm) as a measure related to acidity^6^. All CEST-SAGE-EPI and CEST-EPI data were corrected for motion by using rigid transformation (*mcflirt*; Functional Magnetic Resonance Imaging of the Brain Software Library, Oxford, UK; http://www.fmrib.ox.ac.uk/fsl/) and corrected for B_0_ inhomogeneities by using a z-spectra-based k-means clustering and Lorentzian fitting algorithm^37^. Then, an integral with a width of 0.4 ppm was calculated around both the − 3.0 and + 3.0 ppm spectral points (− 3.2 to − 2.8 ppm and + 2.8 to + 3.2 ppm, respectively). They were coupled with the corresponding S_0_ image to quantify the asymmetry in the magnetization transfer ratio (MTR_asym_) at 3.0 ppm, a measure related to pH^6^, as defined by the following equation: MTR_asym_(3.0 ppm) = S(− 3.0 ppm)/S_0_ − S(+ 3.0 ppm)/S_0_, where S(ω) is the signal of bulk water obtained after the saturation pulse with offset frequency ω, and S_0_ is the signal obtained without application of the saturation pulse. For CEST-SAGE-EPI data, the first and second gradient echoes were averaged for the MTR_asym_ at 3.0 ppm to augment the available signal-to-noise. Creation of MTR_asym_ at 3.0-ppm maps was performed with MatLab (release 2018a, MathWorks) using in-house programs. All MR images were registered to the corresponding three-dimensional CE-T1WI and interpolated to a 1-mm isovoxel for each patient by using a six-degree-of-freedom rigid transformation and a mutual information cost function with FSL software (*flirt*; Functional Magnetic Resonance Imaging of the Brain Software Library). Three mutually exclusive regions of interest (ROIs) were defined using a semi-automated thresholding method^38^ with the Analysis of Functional NeuroImages software (NIMH Scientific and Statistical Computing Core; Bethesda, MD, USA; https://afni.nimh.nih.gov). They included (a) a contrast-enhancing tumor and (b) central necrosis defined by T1-weighted digital subtraction maps; and (c) the T2 hyperintense regions on T2-weighted FLAIR images (non-enhancing tumor), excluding areas of contrast enhancement and necrosis. These mutually exclusive ROIs were combined to be used as a tumor ROI. In this study, four images, including CE-T1WI and FLAIR images, MTR_asym_ at 3.0 ppm maps, and ADC maps, were used for machine learning. The signal intensity/quantitative value was z-score normalized.

### Unsupervised two-level clustering approach

**Figure 6:**
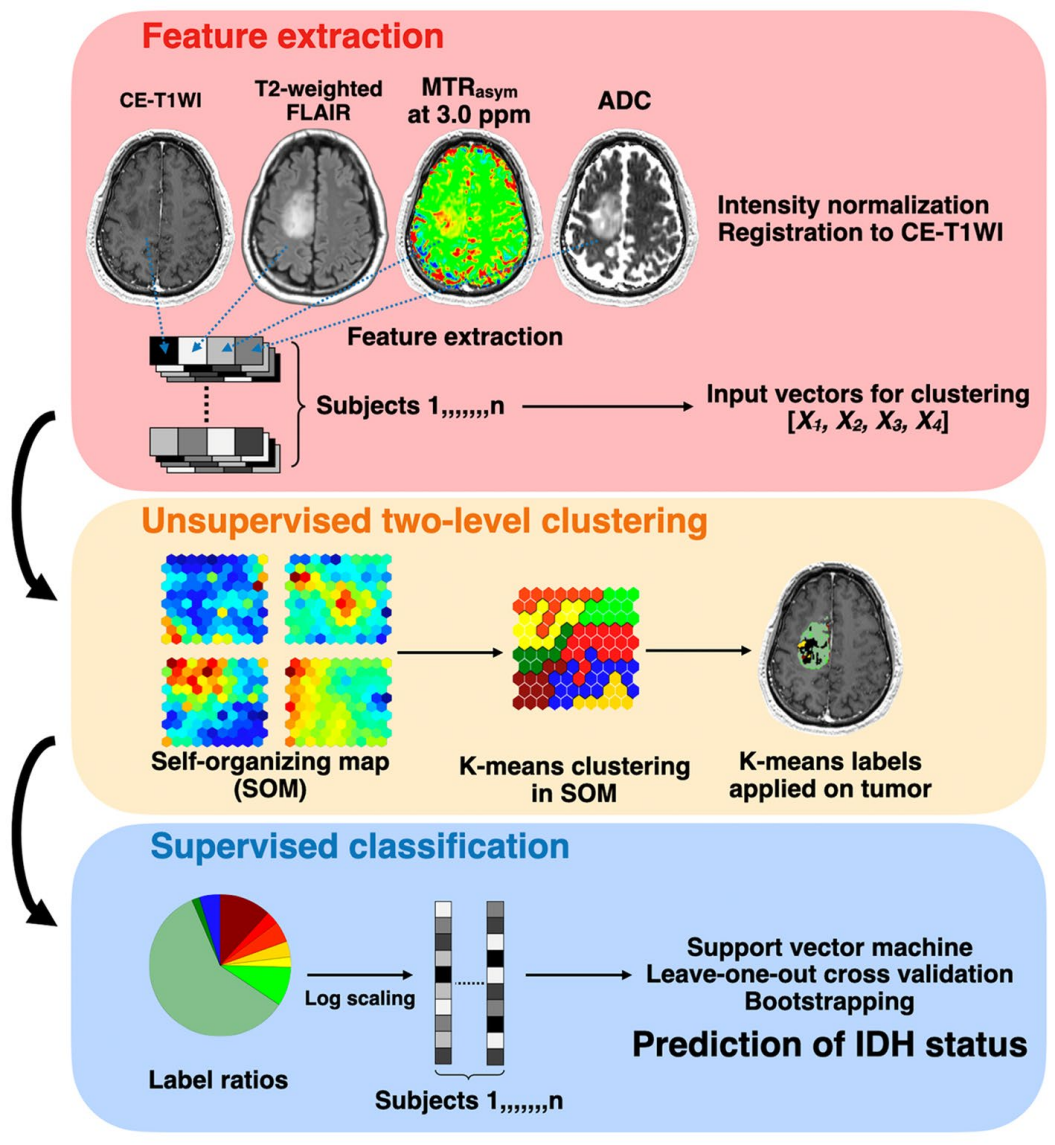
Graphical overview of the processing pipeline.

The overview of the processing pipeline is illustrated in Fig. 6. Features for unsupervised clustering were extracted from voxels on the four parameters of normalized original images every 64 (4 × 4 × 4) voxels within the binary whole-brain mask image obtained with FSL’s brain extraction tool (*bet*; Functional Magnetic Resonance Imaging of the Brain Software Library). The extracted features from four different images of all subjects were stacked and used as input vectors (dimension: 4 × 112075) for voxel-based clustering. A two-level clustering approach was applied using a batch-learning SOM and the K-means algorithm for unsupervised clustering^39,40^. A large number of input vectors was clustered into protoclusters (weighted vectors). Next, the protoclusters were classified into the expected number of clusters by a K-means algorithm using the weighted vectors of each protocluster. Vesanto and Alhoniemi^41^ suggested that the number of protoclusters N was determined as N = k^2^_max_, where k_max_ was the maximum number of clusters for two-level clustering. Vijayakumar et al.^42^. and Inano et al.^39,43^. used SOM for segmentation of brain tumor and grading of gliomas, respectively, on MRI with pre-defined 400 (20 × 20) protoclusters. According to these previous reports and formula, 400 (20 × 20) protoclusters seem to be acceptable for the current study. We have little prior knowledge about the appropriate number of K, and it may differ according to what problem to solve using the clustered images. On the basis of previous studies^39,44^, we chose the K-class numbers with K = 4, 6, 8, 10, 12, 16, 20. After unsupervised clustering by SOM followed by the K-means, 400 (20 × 20) protoclusters with K-class label information were generated. The label information of the nearest protocluster was assigned to each voxel on the four intensity-normalized original images within the tumor ROIs. To evaluate the ratios of labels for each K-class within tumor ROIs, the common logarithmic value of the ratio was calculated by the formula: log_10_ (p + 10^−2^), where p is the ratio of each label (%). Then, the ratios of each K-class label for all participants were applied as input features (dimension: K-class × 69 [subjects]) to the subsequent support vector machine (SVM) classification. We implemented this two-level clustering algorithm using MATLAB software (R2018a; MathWorks, Natick, MA, USA).

### Classification using SVM

By applying the ratios of each K-class label as extracted features, a linear SVM was chosen as a classifier to differentiate IDH mutation status, and the hyperparameters of the linear SVM with a two-step grid search technique were optimized, as previously described^39^. A leave-one-out cross-validation (LOOCV) strategy was carried out to assess the classification performance, allowing us to use most of the data for training. The decision function derived from the training datasets was used to classify or calculate a decision value for the test participant. After the LOOCV, the AUC of receiver operating characteristic (ROC) curves, accuracy, sensitivity, specificity, precision, recall, and F1 score were calculated. Additionally, the patients’ age was included in the SVM analysis to investigate if it improved prediction performance. We used MATLAB software (R2018a; MathWorks, Natick, MA, USA) to implement a linear kernel SVM and LOOCV strategy.

### Biopsy acquisition and immunohistochemistry (IHC)

For a group of the participants included in this study, two to four MRI targets as spheres of 5-mm diameter were defined for each patient prior to surgery, and were used as biopsy targets. These targets were selected based on the MTR_asym_ at 3.0 ppm with high/low values. Biopsy targets were transferred to intraoperative navigation software (Brainlab, Munich, Germany). Standard of care tumor resection was carried out while acquiring tissues corresponding to biopsy targets under intraoperative neuronavigation guidance. The MRI labels used for IDH prediction were categorized as *M*, *N*, and *W* if the logarithmic ratio of the label was significantly higher in the IDH mutant than in the wild-type gliomas, if the logarithmic ratio of the label was not significantly different between IDH mutant and wild-type gliomas, and if the logarithmic ratio of the label was significantly lower in IDH mutant than wild-type gliomas, respectively. The target ROI was assigned to the category that occupied the majority of the ROI. IHC analysis was performed using antibodies for HIF1a, GLUT3, HK2, MCT1, LDHA, and Ki67. The details of IHC analysis are described in Supplementary Method 1.

### Statistical analysis

To determine if the classification performances were significantly different among the different K-classes (K = 4, 6, 8, 10, 12, 16, 20), we performed SVM classification in each K-class 100 times by using a bootstrap technique and then analyzed the differences by a one-way analysis of variance followed by Tukey’s multiple-comparison tests. To compare the log-ratio values of each label in the K-class with the best classification performance between IDH mutant and wild-type gliomas, a Mann–Whitney U test with the Benjamini–Hochberg method for multiple-comparison corrections was used. Histological measurements of IDH mutant and wild-type gliomas were compared between categories *M*, *N*, and *W*, using the Kruskal–Wallis test and Dunn's test for the multiple-comparison corrections. Further, histological measurements were compared between IDH mutant and wild-type gliomas in each category by using the Mann–Whitney U test with the Benjamini–Hochberg method for multiple comparisons to determine whether each category showed similar metabolic features for both IDH mutant and wild-type gliomas. We also compared histological measurements between IDH mutant and wild-type gliomas based on their pathological diagnoses, without considering MRI labels, using the Mann–Whitney U test. Statistical significance was defined as P < 0.05. All statistical analyses were performed on MATLAB software (R2018a; MathWorks, Natick, MA, USA).

### Ethical issue

This retrospective study was approved by the “Medical IRB #2” at the University of California Los Angeles in accordance with the Helsinki Declaration of 1964. All patients provided informed written consent to have advanced imaging and medical information included in our IRB-approved research database according to IRB#14-001261 or IRB#10-000655 approved by Medical IRB #2 at the University of California Los Angeles. Out of the 69 patients, 14 were prospectively included in study IRB#14-001261, which involved surgical validation of CEST imaging method. The other 55 patients received CEST scan as part of the brain tumor standard-of-care MRI protocol in our institute. The usage of their imaging data was approved by the retrospective study protocol IRB#10-000655.

## Supplementary Information

Supplementary Information.

## Data Availability

Datasets analyzed during this study are available from the corresponding author on request. The actual raw imaging data from our patients are completely restricted due to legal and ethical restrictions on sharing these data because of potentially identifying or sensitive patient information, imposed by federal law and the ethics committee of the University of California, Los Angeles.
