# Supplementary material for: Visualization of tumor heterogeneity and prediction of isocitrate dehydrogenase mutation status for human gliomas using multiparametric physiologic and metabolic MRI
Source: Sci Rep. 2022 Jan 20;12:1078. doi: 10.1038/s41598-022-05077-2 (PMC8776874; doi:10.1038/s41598-022-05077-2)
Supplement: Supplementary file 1 — Supplementary Information. [file 41598_2022_5077_MOESM1_ESM.docx]

**Supplementary data**

**Visualization of tumor heterogeneity and prediction of isocitrate dehydrogenase mutation status for human gliomas by using multiparametric physiologic and metabolic MRI**

Akifumi Hagiwara, MD, PhD,^1,2,3^ Hiroyuki Tatekawa, MD, PhD,^1,2,4^ Jingwen Yao, MS,^1,2,5^ Catalina Raymond, MS,^1,2^ Richard Everson, MD,^6^ Kunal Patel, MD,^6^ Sergey Mareninov, BS,^7^ William H. Yong, MD,^7^ Noriko Salamon, MD,^2^ Whitney B. Pope, MD,^2^ Phioanh L. Nghiemphu, MD,^8,9^ Linda M. Liau, MD,^6^ Timothy F. Cloughesy, MD,^8,9^ and Benjamin M. Ellingson, PhD^1,2,5,8,10*^

1. UCLA Brain Tumor Imaging Laboratory (BTIL), Center for Computer Vision and Imaging Biomarkers, University of California, Los Angeles, Los Angeles, CA, USA

2. Department of Radiological Sciences, David Geffen School of Medicine, University of California, Los Angeles, Los Angeles, CA, USA

3. Department of Radiology, Juntendo University School of Medicine, Tokyo, Japan

4. Department of Diagnostic and Interventional Radiology, Osaka City University Graduate School of Medicine, Osaka, Japan

5. Department of Bioengineering, Henry Samueli School of Engineering and Applied Science, University of California Los Angeles, Los Angeles, CA, USA

6. Department of Neurosurgery, David Geffen School of Medicine, University of California, Los Angeles, Los Angeles, CA, USA

7. Department of Pathology, David Geffen School of Medicine, University of California Los Angeles, Los Angeles, CA, USA

8. UCLA Neuro-Oncology Program, University of California, Los Angeles, Los Angeles, CA, USA

9. Department of Neurology, David Geffen School of Medicine, University of California Los Angeles, Los Angeles, CA, USA

10. Department of Psychiatry and Biobehavioral Sciences, David Geffen School of Medicine, University of California Los Angeles, Los Angeles, CA, USA

*** Corresponding author**

Benjamin M. Ellingson, Ph.D.

Director, UCLA Brain Tumor Imaging Laboratory (BTIL)

Professor of Radiology, Biomedical Physics, Psychiatry, and Bioengineering

Departments of Radiological Sciences and Psychiatry

David Geffen School of Medicine

University of California, Los Angeles

924 Westwood Blvd., Suite 615,

Los Angeles, CA 90024

Phone: +1-310-481-7572

Fax: +1-310-794-2796

E-mail: bellingson@mednet.ucla.edu

**Supplementary Method 1**

*Immunohistochemistry (IHC)*

IHC analysis was performed on 5-μm formalin-fixed, paraffin-embedded tissue sections with a customizable IHC detection system. This all-in-one detection system contained Antigen Retrieval Stock Solution (100x), Antibody Diluent, Peroxidase Block, Protein Block, Horseradish Peroxidase Polymer Conjugated Secondary Antibody, DAB Chromogen, DAB Substrate Buffer, and Hematoxylin and Bluing Solution. Heat-induced antigen retrieval was accomplished with Antigen Retrieval Buffer (pH 6.0) in a Decloaking Chamber at 95°C for 30 min (Biocare Medical, Concord, CA, USA). Then, tissue sections were treated with Peroxidase Block and with Protein Block to reduce nonspecific background staining. Primary antibodies for hypoxia-inducible factor 1-alpha (HIF1a) (Sigma/Aldrich, HPA001275, 1:200), glucose transporter 3 (GLUT3) (Sigma-Aldrich, HPA006539, 1:200), hexokinase 2 (HK2) (Sigma-Aldrich, HPA028587, 1:100), monocarboxylate transporter 1 (MCT1) (Sigma-Aldrich, SAB2702323, 1:200), lactic dehydrogenase A (LDHA) (Abcam, Ab47010, 1:500), and Ki67 (Sigma/Aldrich, 275R-15, 1:100) were applied for 60 min followed by detection with Horseradish Peroxidase Polymer Conjugated Secondary Antibody (anti-Rabbit or anti-Mouse). Subsequent immunodetection was completed using the DAB Chromogen and counterstaining was performed with Hematoxylin and Bluing Solution. The tissue slides were scanned on a digital slide scanner (Aperio CS2; Aperio Technologies). The percentage of positive cells was calculated as the ratio of the number of positive cells to total cells in a tissue section by using a semi-automated positive cell detection algorithm implemented in QuPath ver. 0.2.0-m8 (<https://qupath.github.io/>) [1].

**References**

1. Bankhead P, Loughrey MB, Fernandez JA, Dombrowski Y, McArt DG, Dunne PD, McQuaid S, Gray RT, Murray LJ, Coleman HG, James JA, Salto-Tellez M, Hamilton PW (2017) QuPath: Open source software for digital pathology image analysis. Sci Rep 7: 16878 doi:10.1038/s41598-017-17204-5

|  | | **Supplementary Table 1** Detailed patient demographics and information regarding imaging and imaging-guided biopsy | | | | | | | | | |
| --- | --- | --- | --- | --- | --- | --- | --- | --- | --- | --- | --- |
| ID | Sex | Age | WHO grade | IDH mutation status | 1p/19q codeletion status | Scanner | CEST | Diffusion | Biopsy | Biopsy category |  |
| 1 | Male | 22 | II | Mutant | Non-codeleted | Prisma | CEST-SAGE-EPI | DTI |  |  |  |
| 2 | Male | 26 | II | Mutant | Codeleted | Prisma | CEST-EPI | DTI | Performed | M/M |  |
| 3 | Male | 32 | II | Mutant | Codeleted | Skyra | CEST-SAGE-EPI | DTI |  |  |  |
| 4 | Male | 33 | II | Mutant | Codeleted | Prisma | CEST-SAGE-EPI | DTI |  |  |  |
| 5 | Male | 34 | II | Mutant | Non-codeleted | Trio | CEST-EPI | DTI |  |  |  |
| 6 | Male | 38 | II | Mutant | Codeleted | Prisma | CEST-SAGE-EPI | DTI | Performed | M/M/N/N |  |
| 7 | Male | 38 | II | Mutant | Codeleted | Skyra | CEST-SAGE-EPI | DTI |  |  |  |
| 8 | Male | 40 | II | Mutant | Codeleted | Skyra | CEST-SAGE-EPI | DTI |  |  |  |
| 9 | Male | 56 | II | Mutant | Non-codeleted | Trio | CEST-EPI | DTI |  |  |  |
| 10 | Male | 59 | II | Mutant | Non-codeleted | Skyra | CEST-SAGE-EPI | DTI |  |  |  |
| 11 | Male | 61 | II | Mutant | Codeleted | Skyra | CEST-SAGE-EPI | DTI |  |  |  |
| 12 | Male | 78 | II | Mutant | Non-codeleted | Prisma | CEST-SAGE-EPI | DTI |  |  |  |
| 13 | Female | 33 | II | Mutant | Non-codeleted | Trio | CEST-EPI | DTI |  |  |  |
| 14 | Female | 38 | II | Mutant | Non-codeleted | Prisma | CEST-EPI | DTI | Performed | M/M |  |
| 15 | Female | 38 | II | Mutant | Codeleted | Skyra | CEST-SAGE-EPI | DTI |  |  |  |
| 16 | Female | 48 | II | Mutant | Codeleted | Prisma | CEST-SAGE-EPI | DTI |  |  |  |
| 17 | Female | 50 | II | Mutant | Non-codeleted | Prisma | CEST-SAGE-EPI | DTI |  |  |  |
| 18 | Female | 52 | II | Mutant | Codeleted | Prisma | CEST-SAGE-EPI | DTI |  |  |  |
| 19 | Female | 60 | II | Mutant | Codeleted | Prisma | CEST-SAGE-EPI | DTI |  |  |  |
| 20 | Male | 26 | III | Mutant | Non-codeleted | Prisma | CEST-SAGE-EPI | DTI |  |  |  |
| 21 | Male | 29 | III | Mutant | Non-codeleted | Prisma | CEST-SAGE-EPI | DWI | Performed | M/M/M/N |  |
| 22 | Male | 49 | III | Mutant | Codeleted | Prisma | CEST-EPI | DWI | Performed | M/M |  |
| 23 | Male | 49 | III | Mutant | Non-codeleted | Prisma | CEST-SAGE-EPI | DTI | Performed | M/N/N/N |  |
| 24 | Male | 59 | III | Mutant | Non-codeleted | Prisma | CEST-SAGE-EPI | DTI |  |  |  |
| 25 | Male | 65 | III | Mutant | Codeleted | Prisma | CEST-SAGE-EPI | DTI |  |  |  |
| 26 | Female | 28 | III | Mutant | Non-codeleted | Prisma | CEST-EPI | DTI |  |  |  |
| 27 | Female | 30 | III | Mutant | Non-codeleted | Prisma | CEST-SAGE-EPI | DTI |  |  |  |
| 28 | Female | 39 | III | Mutant | Codeleted | Prisma | CEST-SAGE-EPI | DTI |  |  |  |
| 29 | Female | 40 | III | Mutant | Non-codeleted | Trio | CEST-EPI | DTI |  |  |  |
| 30 | Female | 52 | III | Mutant | Non-codeleted | Prisma | CEST-SAGE-EPI | DTI |  |  |  |
| 31 | Female | 58 | III | Mutant | Non-codeleted | Skyra | CEST-SAGE-EPI | DTI |  |  |  |
| 32 | Male | 24 | IV | Mutant | Non-codeleted | Skyra | CEST-SAGE-EPI | DTI |  |  |  |
| 33 | Male | 62 | II | Wild type | – | Skyra | CEST-SAGE-EPI | DTI | Performed | N/N/W |  |
| 34 | Male | 63 | II | Wild type | – | Trio | CEST-EPI | DTI |  |  |  |
| 35 | Male | 21 | III | Wild type | – | Prisma | CEST-SAGE-EPI | DWI |  |  |  |
| 36 | Male | 53 | III | Wild type | – | Prisma | CEST-SAGE-EPI | DTI | Performed | M/M/M |  |
| 37 | Male | 59 | III | Wild type | – | Skyra | CEST-SAGE-EPI | DTI | Performed | N/N/N |  |
| 38 | Male | 68 | III | Wild type | – | Prisma | CEST-SAGE-EPI | DTI |  |  |  |
| 39 | Male | 68 | III | Wild type | – | Prisma | CEST-SAGE-EPI | DTI |  |  |  |
| 40 | Female | 34 | III | Wild type | – | Trio | CEST-EPI | DTI |  |  |  |
| 41 | Female | 59 | III | Wild type | – | Prisma | CEST-SAGE-EPI | DTI |  |  |  |
| 42 | Female | 59 | III | Wild type | – | Skyra | CEST-SAGE-EPI | DTI |  |  |  |
| 43 | Female | 70 | III | Wild type | – | Prisma | CEST-EPI | DTI | Performed | N/N/N |  |
| 44 | Male | 35 | IV | Wild type | – | Trio | CEST-EPI | DTI | Performed | W/W/W |  |
| 45 | Male | 38 | IV | Wild type | – | Trio | CEST-EPI | DWI |  |  |  |
| 46 | Male | 42 | IV | Wild type | – | Trio | CEST-EPI | DTI |  |  |  |
| 47 | Male | 48 | IV | Wild type | – | Trio | CEST-EPI | DTI |  |  |  |
| 48 | Male | 51 | IV | Wild type | – | Skyra | CEST-SAGE-EPI | DWI |  |  |  |
| 49 | Male | 51 | IV | Wild type | – | Skyra | CEST-SAGE-EPI | DTI |  |  |  |
| 50 | Male | 52 | IV | Wild type | – | Trio | CEST-EPI | DTI |  |  |  |
| 51 | Male | 53 | IV | Wild type | – | Prisma | CEST-SAGE-EPI | DTI |  |  |  |
| 52 | Male | 56 | IV | Wild type | – | Skyra | CEST-SAGE-EPI | DTI |  |  |  |
| 53 | Male | 59 | IV | Wild type | – | Prisma | CEST-SAGE-EPI | DTI | Performed | W/W/W/W |  |
| 54 | Male | 59 | IV | Wild type | – | Prisma | CEST-SAGE-EPI | DTI |  |  |  |
| 55 | Male | 60 | IV | Wild type | – | Trio | CEST-EPI | DTI |  |  |  |
| 56 | Male | 61 | IV | Wild type | – | Prisma | CEST-SAGE-EPI | DTI |  |  |  |
| 57 | Male | 61 | IV | Wild type | – | Trio | CEST-EPI | DTI |  |  |  |
| 58 | Male | 61 | IV | Wild type | – | Skyra | CEST-SAGE-EPI | DTI | Performed | N/W/W |  |
| 59 | Male | 63 | IV | Wild type | – | Trio | CEST-EPI | DTI |  |  |  |
| 60 | Male | 67 | IV | Wild type | – | Skyra | CEST-SAGE-EPI | DTI |  |  |  |
| 61 | Male | 69 | IV | Wild type | – | Trio | CEST-EPI | DTI |  |  |  |
| 62 | Male | 70 | IV | Wild type | – | Prisma | CEST-EPI | DTI | Performed | M/N/W/W |  |
| 63 | Male | 71 | IV | Wild type | – | Trio | CEST-EPI | DTI |  |  |  |
| 64 | Male | 75 | IV | Wild type | – | Trio | CEST-EPI | DTI |  |  |  |
| 65 | Female | 19 | IV | Wild type | – | Trio | CEST-EPI | DTI |  |  |  |
| 66 | Female | 60 | IV | Wild type | – | Trio | CEST-EPI | DTI |  |  |  |
| 67 | Female | 62 | IV | Wild type | – | Skyra | CEST-SAGE-EPI | DTI |  |  |  |
| 68 | Female | 64 | IV | Wild type | – | Trio | CEST-EPI | DWI |  |  |  |
| 69 | Female | 80 | IV | Wild type | – | Trio | CEST-EPI | DTI |  |  |  |

Note: Biopsy category M includes labels typical for IDH mutant gliomas, whereas category W includes labels typical for IDH wild-type gliomas. Category N includes labels not typical either for IDH mutant nor wild-type gliomas.

| **Supplementary Table 2** Prediction performances of all K-classes | | | | | |  |  |
| --- | --- | --- | --- | --- | --- | --- | --- |
| Number of K-class | 4 | 6 | 8 | 10 | 12 | 16 | 20 |
| AUC | 0.78 [0.77–0.79] | 0.74 [0.73–0.76] | 0.92 [0.92–0.93] | 0.94 [0.94–0.95] | 0.90 [0.89–0.91] | 0.93 [0.92–0.94] | 0.93 [0.92–0.93] |
| Accuracy | 0.72 [0.71–0.74] | 0.70 [0.69–0.72] | 0.87 [0.86–0.88] | 0.91 [0.90–0.92] | 0.86 [0.85–0.86] | 0.90 [0.89–0.91] | 0.88 [0.87–0.89] |
| Sensitivity | 0.66 [0.63–0.68] | 0.62 [0.60–0.65] | 0.85 [0.83–0.86] | 0.90 [0.89–0.91] | 0.82 [0.81–0.84] | 0.88 [0.86–0.89] | 0.88 [0.87–0.89] |
| Specificity | 0.76 [0.75–0.78] | 0.75 [0.73–0.77] | 0.88 [0.87–0.89] | 0.91 [0.90–0.92] | 0.88 [0.87–0.89] | 0.91 [0.90–0.92] | 0.88 [0.87–0.89] |
| Precision | 0.71 [0.69–0.72] | 0.68 [0.66–0.71] | 0.86 [0.85–0.87] | 0.90 [0.89–0.91] | 0.85 [0.84–0.86] | 0.89 [0.88–0.90] | 0.86 [0.85–0.88] |
| Recall | 0.66 [0.63–0.68] | 0.62 [0.60–0.65] | 0.85 [0.83–0.86] | 0.90 [0.89–0.91] | 0.82 [0.81–0.84] | 0.88 [0.86–0.89] | 0.88 [0.87–0.89] |
| F1-score | 0.68 [0.66–0.70] | 0.65 [0.63–0.67] | 0.85 [0.84–0.87] | 0.90 [0.89–0.91] | 0.84 [0.82–0.85] | 0.88 [0.87–0.90] | 0.87 [0.86–0.88] |

Note: Values are shown as mean and 95% confidence intervals.


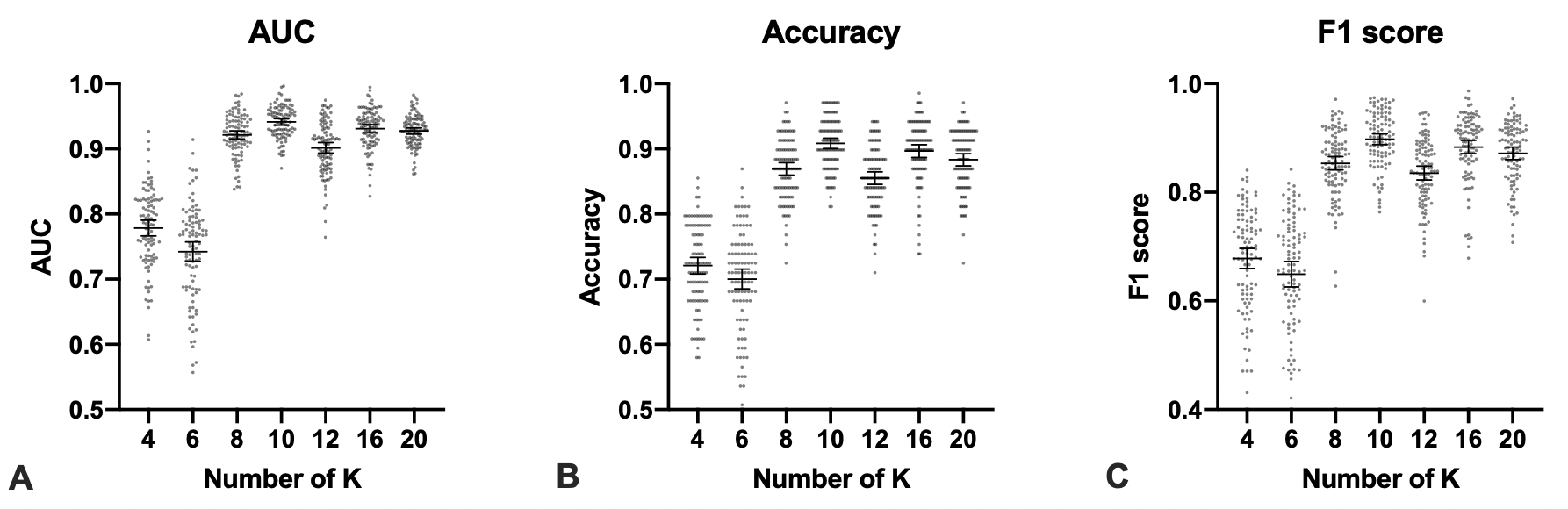


**Supplemental Figure 1.** A) The AUC, B) accuracy, and C) F1 score to predict IDH mutation status for K = 4, 6, 8, 10, 12, 16, 20 using 100 bootstraps are shown. Bars indicate mean values with 95% confidence interval. The 10-class clustering shows the highest AUC, accuracy, and F1-score.
